# Deciphering macrophage molecular responses to *Porphyromonas gingivalis* outer membrane vesicles through combined immunoassay and Raman spectroscopic analyses

**DOI:** 10.3389/fmicb.2026.1803640

**Published:** 2026-05-29

**Authors:** Yoshiyuki Matsuo, Aoi Son, Sara Gentile, Riccardo Bernola, Takashi Yamashita, Saki Ikegami, Lucia Sileo, Luna Ardondi, Toru Kariu, Barbara Zavan, Wenliang Zhu, Davide Franceschini, Elisa Bevilacqua, Konstantinos Koutsikos, Leonardo Rigon, Yuji Kamioka, Daniela Secci, Paolo Pinton, Giuseppe Pezzotti

**Affiliations:** 1 Central Research Center, Institute of Biomedical Science, Kansai Medical University, Hirakata, Japan; 2 Biomedical Engineering Center, Kansai Medical University, Hirakata, Japan; 3 Department of Chemistry and Technologies of Drugs, Sapienza University of Rome, Rome, Italy; 4 Ceramic Physics Laboratory, Kyoto Institute of Technology, Kyoto, Japan; 5 Department of Translational Medicine, University of Ferrara, Ferrara, Italy; 6 Department of Food and Fermentation Science, Beppu University, Beppu, Japan; 7 Biomarker Disease Laboratory, IRCCS San Camillo Hospital, Venice, Italy; 8 Padova Neuroscience Center (PNC), University of Padua, Padua, Italy; 9 Department of Molecular Genetics, Institute of Biomedical Science, Kansai Medical University, Hirakata, Japan; 10 Laboratory for Technologies of Advanced Therapies (LTTA), Department of Medical Sciences, University of Ferrara, Ferrara, Italy; 11 International Center for Biomedical Industrial Promotion, Kansai Medical University, Osaka, Japan; 12 Department of Immunology, Graduate School of Medical Science, Kyoto Prefectural University of Medicine, Kyoto, Japan; 13 Department of Orthopedic Surgery, Tokyo Medical University, Tokyo, Japan; 14 Department of Molecular Science and Nanosystems, Ca’ Foscari University of Venice, Venice, Italy; 15 Department of Orthopaedic Surgery, Graduate School of Medicine, Mie University, Tsu, Japan; 16 Teikyo University Institute of Medical Mycology (TIMM), Graduate School of Medicine, Teikyo University, Tokyo, Japan; 17 Department of Bacteriology, Graduate School of Biomedical & Health Sciences, Hiroshima University, Hiroshima, Japan

**Keywords:** macrophage, mitochondrial function, outer membrane vesicles (OMV), *Porphyromonas gingivalis*, Raman spectroscopy

## Abstract

Outer membrane vesicles released by Porphyromonas gingivalis (Pg-OMVs) are emerging as critical mediators of periodontal and systemic pathology. Published literature has so far associated the presence of OMVs to an increased risk of cerebrovascular and neurodegenerative diseases, but the molecular-scale mechanisms underlying their interactions with immune cells remain insufficiently understood and characterized. Here, we employ high-resolution Raman spectroscopy in addition to established immunoassays to quantitatively decode the real-time chemical reprogramming induced by Pg-OMVs in macrophages. Our results reveal early and pronounced alterations in lipid composition, including significant increases in sphingomyelin- and ceramide-associated vibrational signatures, paralleled by perturbations in cholesterol-rich membrane domains. These lipidomic changes coincide with Raman markers of mitochondrial dysfunction, as characterized by reduced cytochrome c oxidation signals, indicating a shift toward a more reduced redox state. We further identify time-resolved modifications of glycogen-associated bands, implicating metabolic rewiring and inflammatory activation. Together, these spectral fingerprints demonstrate how Pg-OMVs orchestrate a coordinated disruption of membrane architecture, intracellular lipid homeostasis, and mitochondrial function. Raman findings provide a non-destructive, label-free molecular atlas of OMV-driven macrophage dysregulation and establish Raman spectroscopy as a powerful platform for quantitatively elucidating host–pathogen interactions with direct relevance to oral-systemic health and chronic inflammatory disease.

## 1 Introduction

Periodontitis is a chronic inflammatory disease of the periodontium that implicates oral microbial dysbiosis, host immune dysregulation, and tissue destruction, including alveolar bone resorption (Uemura et al., 2022; How et al., 2016). Among the implicated pathogens, *Porphyromonas gingivalis* (*Pg*) has been identified as a keystone bacterium that contributes to the initiation and progression of periodontitis through multiple virulence mechanisms, modulation of the host immune system, and interaction with other members of the oral microbiome. *Pg* is a gram-negative, anaerobic bacterium that resides in subgingival biofilms and generates a wide array of virulence factors including lipopolysaccharide with atypical tetra- and penta-acylated lipids, gingipain proteinases, fimbriae, and outer membrane proteins (How et al., 2016; Coats et al., 2025; Rocha et al., 2021; Fleetwood et al., 2017; Wu et al., 2025). One of the increasingly recognized mechanisms by which *Pg* influences the host is the generation and release of outer membrane vesicles (OMVs) filled up with a variety of toxins (Wu et al., 2025; Fan et al., 2023; Mao et al., 2023). OMVs are nano-sized, bilayered vesicles shed from the outer membrane of Gram-negative bacteria, containing periplasmic and outer-membrane proteins, lipids (including phospholipids and sphingolipids), nucleic acids, and other virulence components (Schwechheimer and Kuehn, 2015; Zhao et al., 2025). OMVs represent a formidable vehicle for long-distance delivery of bacterial molecules, since they can traverse barriers and modulate host cell responses. Within the oral context, *Pg*-OMVs are capable of binding to and swiftly entering host cells, delivering virulence factors and influencing immune responses, epithelial barrier integrity, and even distal systemic effects (Wu et al., 2025; Mahendrarajan et al., 2025; Gui et al., 2016).

In macrophages, the key innate immune cells present in the periodontal microenvironment, *Pg*-OMVs have been shown to induce distinct responses compared to whole bacteria (Wang et al., 2025; Okamura et al., 2021). For instance, macrophages stimulated with *Pg*-OMVs have been reported to produce elevated levels of pro-inflammatory cytokines and nitric oxide, and display metabolic rewiring from oxidative phosphorylation to glycolysis, increased mitochondrial oxygen radicals, and induction of inflammasome activation (Zhang S. et al., 2025; Zhang Z. et al., 2025). Macrophages exposed to OMVs were also found to undergo a cell death characteristic of pyroptosis (Fleetwood et al., 2017; Resta et al., 2024; Lin et al., 2022; Cecil et al., 2017). The above body of pro-inflammatory characteristics suggests that *Pg*-OMVs are not simply by-products, but active mediators of host-pathogen cross talk. The OMV cargo from *Pg* is indeed a complex ensemble, which includes a variety of diverse virulence molecules with inflammasome activation and immune evasion potential; a “decoy” strategy that allows *Pg* to redirect or modulate host responses (Castro-Vargas et al., 2024). Moreover, *Pg*-OMVs carry ceramide and sphingolipids that dampen macrophage inflammatory responses, effectively modulating the innate immune signalling network (Makkawi et al., 2017).

Given the central role of macrophages in host defense, inflammation, tissue homeostasis and resolution of infection, their interactions with OMVs from *Pg* deserve detailed molecular-scale investigations. Macrophages in the periodontal lesion are continuously exposed to bacterial products and respond via phagocytosis, cytokine release, reactive oxygen/nitrogen species production, metabolic adaptation, activation of inflammasomes, and modulation of phenotype. Dysregulation of this response contributes to persistent inflammation, tissue damage, alveolar bone loss, and systemic comorbidities (e.g., Alzheimer’s disease) linked epidemiologically to periodontitis (Afzoon et al., 2023; Guo et al., 2024; Zhang et al., 2021; Nara et al., 2021; Liu et al., 2024). In particular, this association has been widely documented in cerebrovascular diseases, where sustained inflammation may contribute to some of the pathological mechanisms underlying both ischemic and hemorrhagic strokes (e.g., atherosclerosis and vessel fragility, respectively). Similarly, neuroinflammation plays a crucial role in several neurodegenerative diseases, including Parkinson’s and Alzheimer’s, by hampering misfolded protein clearance (Shi and Yong, 2025; El Masri et al., 2024). In this context, deepening our understanding of the complex relationship between Pg-OMVs and inflammation holds remarkable translational potential to integrate the diagnostic and therapeutic frameworks of several pathological conditions, potentially supporting their management from prevention to rehabilitation.

At the molecular scale, many studies have so far relied on biochemical and transcriptomic assays, while few have yet applied spectroscopic approaches to probe *in situ* the chemical microenvironment and molecular architecture of macrophages during OMV challenge.

In the above context, Raman spectroscopy with direct vibrations probes of lipids, proteins, nucleic acids and polysaccharides, could provide a valuable complementary approach. By capturing changes in biochemical composition at sub-cellular resolution, Raman spectroscopy could help reveal key mechanistic signatures of macrophages’ oxidative phosphorylation in response to *Pg*-OMVs exposure, including mitochondrial redox cofactors, lipid-associated membrane restructuration, and dynamic changes in glycosylation levels (Erjavec et al., 2016; Kreiss et al., 2022; Knab et al., 2025; Molnar and Miskolci, 2024; Naumann et al., 2023). Together, selected Raman features could offer a real-time, non-invasive spectral fingerprint reflecting the level of oxidative phosphorylation in living macrophages.

Building upon our previous *in situ* Raman studies of cells and various pathogens (Pezzotti, 2021; Pezzotti et al., 2023; Pezzotti et al., 2024), here we seek to investigate *in vitro* the chemical interaction between *Pg*-OMVs and mouse macrophages, with the goal of specifically locating spectroscopic parameters suitable for the quantification of molecular-scale mechanisms of immune reaction and validate them by means of established biochemical assays. In summary, this study attempts to address the important gap of bridging the biophysical spectroscopic characterization of macrophage molecular composition with the biological immunological consequences of *Pg*-OMVs challenge.

## 2 Materials and methods

### 2.1 Cell culture, OMVs separation, and microscopy

Murine macrophages RAW264.7 were pre-cultured in DMEM (Nacalai Tesque, Kyoto, Japan) supplemented with 10% fetal bovine serum and GlutaMAX^™^ Supplement (35050061; ThermoFisher Scientific, Waltham, MA, United States). Cells were seeded at a density of 1.0 × 10^5^ cells/mL in a 6-well plate (Falcon, Corning Inc., Corning, NY, United States) and incubated in a CO_2_ incubator at 37 °C. After 24 h, cells were stimulated as follows: one well was treated with LPS (100 ng/mL) and IFN-*γ* (20 ng/mL); another well was treated with *Pg*-OMV (0.25 μg/mL) and IFN-γ (20 ng/mL). Cells from each well, in the untreated, LPS/IFN-γ stimulated, and *Pg*-OMV/IFN-γ stimulated states, were detached with trypsin–EDTA at 3, 6 and 24 h post-stimulation. The final cell pellet was then resuspended in 50 μL of PBS (Nacalai Tesque, Kyoto, Japan) for subsequent Raman spectroscopy analysis. *Pg*-OMVs were prepared from *P. gingivalis* culture supernatant as described by Kariu et al. (2019). *P. gingivalis* ATCC33277 was grown in BHI-broth supplemented with hemin and menadione under anaerobic conditions. *Pg*-OMVs were finally resuspended in PBS and stored at –80 °C until use.

Cell morphology was assessed using an EVOS M5000 Imaging System (ThermoFisher Scientific, Waltham, MA, USA). RAW264.7 cells were seeded and cultured under the experimental conditions indicated above. Images were captured after 24 h stimulation, using a 20 × objective lens, and representative fields were recorded for analysis.

### 2.2 Endotoxin quantification

Endotoxin levels of *Pg*-OMVs and purified *E. coli* LPS were determined using the Pierce Chromogenic Endotoxin Quant Kit (Thermo Fisher Scientific, Rockford, IL, United States) according to the manufacturer’s instructions. Samples were analyzed in a 96-well format, and absorbance at 405 nm was recorded with an iMark microplate reader (Bio-Rad, Hercules, CA, United States). The endotoxin standard provided with the kit was used to generate a standard curve, and the results were expressed in endotoxin units (EU/mL).

### 2.3 Measurement of cellular bioenergetics

The oxygen consumption rate (OCR) and extracellular acidification rate (ECAR), which reflect mitochondrial respiration and glycolytic activity, respectively, were measured using a Seahorse XFp Extracellular Flux Analyzer (Agilent Technologies, Santa Clara, CA, United States). RAW264.7 cells were seeded at a density of 2.0 × 10^4^ cells per well in an XFp miniplate and cultured overnight. The cells were stimulated under the indicated experimental conditions at varying concentrations and time points, and the culture medium was replaced with Seahorse XF assay medium (XF DMEM supplemented with 10 mM glucose, 1 mM sodium pyruvate, and 2 mM glutamine). The cells were incubated at 37 °C in a non-CO_2_ incubator for 30 min prior to measurement. OCR and ECAR were recorded over three measurement cycles, each consisting of 3 min of mixing and 3 min of measurement. For the Cell Mito Stress Test, which evaluates multiple components of mitochondrial function, including basal respiration, ATP-linked respiration, maximal respiration, spare respiratory capacity, proton leak, and non-mitochondrial oxygen consumption, OCR was measured under basal conditions, followed by the sequential injection of the following modulators of respiration: 1 μM oligomycin, 1 μM carbonyl cyanide-4-(trifluoromethoxy) phenylhydrazone (FCCP) and 0.5 μM each of rotenone and antimycin A.

In order to evaluate complex III–IV–mediated respiration, rotenone (0.5 μM) was first injected to inhibit complex I, followed by the injection of duroquinol (0.5 mM) to directly donate electrons to complex III. Complex III–IV activity was determined by calculating the increase in OCR upon injection of duroquinol. All measurements of bioenergetics were made on three separate sets of samples (*n* = 3). All reagents used in this study, including their suppliers and stock solution preparations, are listed in Supplementary Table S1.

### 2.4 Quantitative polymerase chain reaction (qPCR)

RAW264.7 cells were seeded at a density of 3.0 × 10^5^ cells per well in a 6-well plate and cultured overnight. The cells were then treated with *P. gingivalis* OMV (1.0 μg/mL) or LPS (0.1 μg/mL) in the presence of IFN-*γ* (0.02 μg/mL) for 16 h. Total RNA was extracted using the Maxwell RSC simplyRNA Cells Kit and the Maxwell RSC Instrument (Promega, Co., Madison, WI, United States). One microgram of total RNA was reverse transcribed into cDNA using the QuantiTect Reverse Transcription Kit (Qiagen, Venlo, Netherlands) according to the manufacturer’s instructions.

Quantitative real-time PCR (qPCR) was performed with the Rotor-Gene SYBR Green PCR kit (Qiagen, Venlo, Netherlands) and gene specific primers. PCR amplification was carried out under the following conditions: initial denaturation at 95 °C for 5 min, followed by 40 cycles of 95 °C for 5 s and 60 °C for 10 s. Relative gene expression was calculated by the ΔΔCt method using Rplp0 gene as an internal control. Measurements were repeated on three separate sets of samples (*n* = 3).

The primers used in this study are listed in Supplementary Table S2.

### 2.5 OMV labeling and imaging

*Pg*-OMVs were fluorescently labeled using the ExoSparkler Exosome Membrane Labeling Kit-Deep Red (Dojindo Molecular Technologies, Kumamoto, Japan) according to the manufacturer’s instructions. RAW264.7 cells were seeded at 3 × 10^5^ cells per 35-mm glass-bottom dish and cultured overnight, followed by incubation with the labeled *Pg*-OMVs (final concentration: 1 μg/mL) for 3 h. Subsequently, the cell membranes were stained with PlasMem Bright Green (Dojindo Molecular Technologies, Kumamoto, Japan) for 10 min to visualize the plasma membrane. The culture medium was replaced with FluoroBrite DMEM (Thermo Fisher Scientific, Rockford, IL, United States) supplemented with 5% fetal bovine serum. Fluorescence images were acquired using a FLUOVIEW FV4000 Confocal Laser Scanning Microscope (Evident, Co., Tokyo, Japan).

### 2.6 Cell Counting Kit-8 (CCK-8) assay

Cell Counting Kit-8 (CCK-8) assay was performed, according to the manufacturer’s protocol (CCK-8; Dojindo Molecular Technologies, Kumamoto, Japan), to assess cell viability after each treatment. RAW264.7 cells were seeded in a 96-well plate (Falcon, Corning Inc., Corning, NY, United States) at a density of 5×10^4^ cells/mL in culture medium (100 μL/well) and incubated in a CO_2_ incubator at 37 °C. After 24 h incubation, RAW264.7 cells were treated with *Pg*-OMV or LPS in combination with interferon-*γ* at different concentrations. After 24 h stimulation, the CCK-8 assay was performed adding the CCK-8 solution to each well and incubating at 37 °C in CO_2_ for two hours.

Absorbance at 450 nm was then measured by means of a microplate reader (iMark; Bio-Rad, Hercules, CA, United States). Also in the case of cell counting assay, measurements were repeated on three separate sets of samples (*n* = 3).

### 2.7 Flow cytometry analysis

The following monoclonal antibodies were used for flow cytometry analysis: ant-CD86-PE (159,203; 1:100; BioLegend, San Diego, CA, United States), anti-I-A/I-E (MHCII)-PE (107,607, 1:100; BioLegend, San Diego, CA, United States). For cell-surface staining, RAW 264.7 cells were first treated with an anti-mouse CD16/32 antibody to block Fc receptors. Cells were then stained with antibodies for 20 min on ice and analyzed at Attune^™^ NxT Acoustic Focusing Cytometer (Invitrogen^™^, ThermoFisher Scientific, Waltham, MA, United States). Data analysis was conducted by means of the FlowJo software (v10.10.0) (Tree Star Inc., Ashland, OR, United States).

### 2.8 Statistical analyses

Data are presented as a mean value ± standard deviation of three independent experiments (*n* = 3). Statistical differences were analyzed by means of one-way ANOVA followed by Dunnett’s multiple-comparison test with the control group as a reference. A value *p* < 0.05 was considered as statistically significant and labeled with an asterisk.

### 2.9 *In situ* Raman spectroscopy measurements

For Raman analysis, a 10-μL aliquot of each cell suspension was pipetted onto a stainless-steel dish. After waiting for the cells to settle for approximately 15 min, spectra were acquired *in situ* using a LabRAM HR800 confocal Raman microscope (Horiba/Jobin-Yvon, Kyoto, Japan). The wavelength of the incoming laser was 532 nm with the laser source operating with a laser power of 70 mW. The spectral resolution of ~1 cm^−1^ was achieved upon analysing the Raman scattered light by a double monochromator connected with an air-cooled charge-coupled device (CCD) detector (Andor DV420-OE322; 1,024 × 256 pixels; Andor Technology, Belfast, Northern Ireland, UK); the grating used in the spectrometer had a resolution of 600 gr/mm. The acquisition time of a single spectrum was 30 s. Two consecutive acquisitions were made at the same spot to minimize noise. The laser spot was focused on the sample through a 10 × optical lens.

In collecting average spectra, sets of ten spectra were collected at different locations over areas of ~2 mm^2^. The raw Raman spectra were first subjected to both solvent and baseline subtraction procedure, as preliminary optimized and standardized according to the asymmetric least square method. Average spectra were then deconvoluted into series of Lorentzian–Gaussian sub-bands using commercial software (LabSpec 4.02, Horiba/Jobin-Yvon, Kyoto, Japan). In performing the above-mentioned deconvolution procedure, a machine-learning approach was applied, which relied on a wide library of Raman spectra from elementary molecules and employed an in-house-built automatic curve-fitting solver, as previously described (Pezzotti, 2021). The Raman ratios that were considered significant for the interpretation of the physiological response of the cells were computed as areal ratios of deconvoluted Lorentzian–Gaussian sub-bands corresponding to selected molecular bonds.

For confirmation, local Raman measurements were also performed by means of an additional Raman device (RAMANtouch, Nanophoton Co., Minoo, Osaka, Japan) with high z-axis spatial resolution, operating in microscopic measurement mode (20 × lens; numerical aperture, NA = 0.45). The excitation source was a 532 nm solid-state laser operating with a power of 40 mW at the sample surface; a 600 grating was used. Spectra were acquired using an exposure time of 10 s and four consecutive acquisitions.

## 3 Experimental results

### 3.1 Biological assays and related statistics

**Figure 1:**
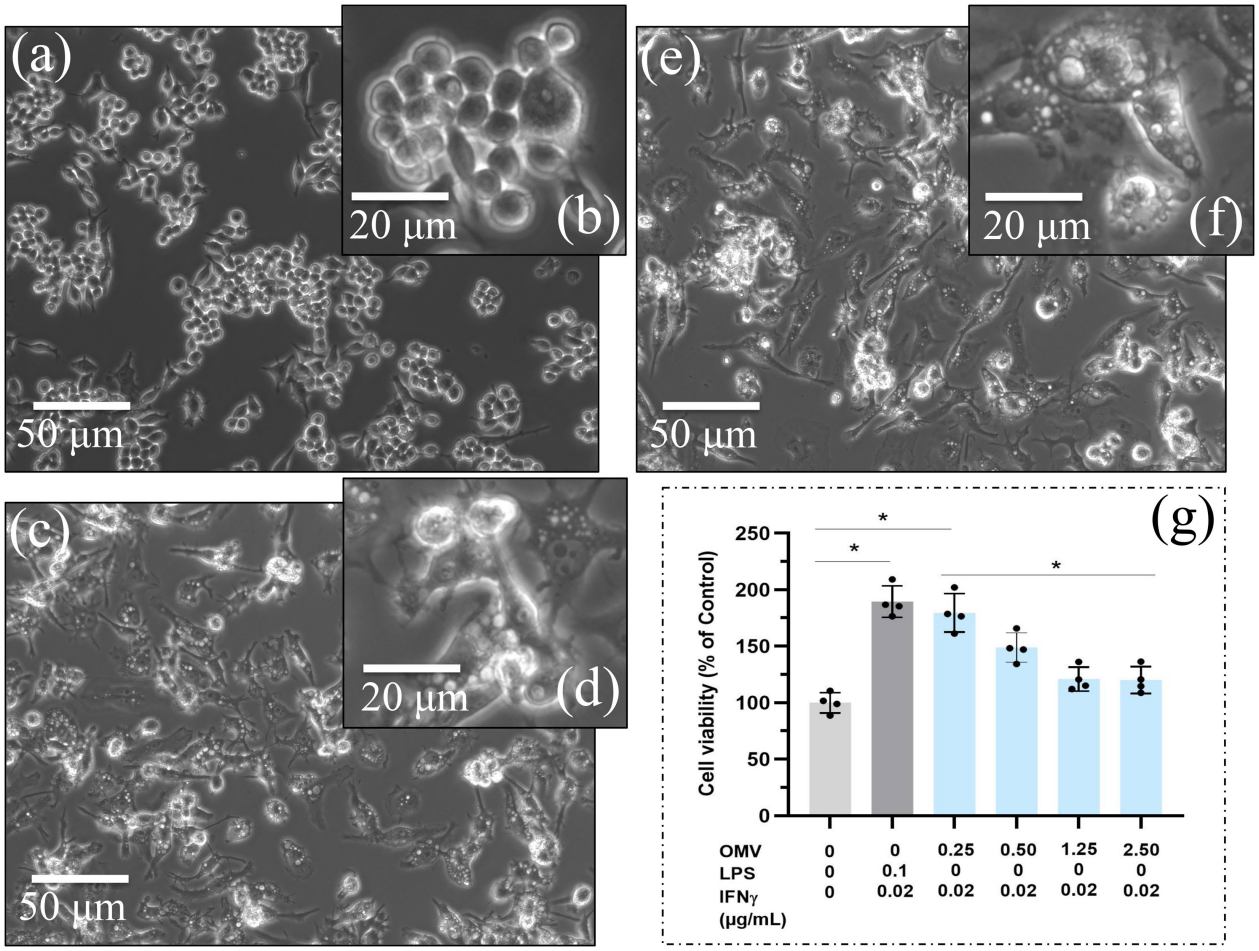
High-resolution optical microscopy images of the morphological alterations to which unexposed macrophages **(a/b)** underwent as a consequence of exposure to LPS **(c/d)** or *Pg*-OMV **(e/f)** (cf. higher magnification images in inset). In **(g)**, a plot is shown with the results of a viability analysis through the CCK-8 assay performed as a function of concentration after 24 h incubation.

Figure 1 shows high-resolution optical microscopy images of the morphological alterations to which macrophages underwent as a consequence of exposure to lipopolysaccharides (LPS) or *Pg*-OMV, in comparison with non-stimulated cells. Exposure to LPS increased cell spreading and promoted a clear transition from a rounded to a flattened configuration (cf. Figures 1a/b,c/d). Irregular stellate appearance, prominent membrane ruffles, and increased vacuolization were the main morphological characteristics, which hinted to an extensive cytoskeletal rearrangement. Very similar cell-morphology characteristics were recorded in the case of stimulation with *Pg*-OMVs (cf. Figures 1e/f), with elongated spindle-like morphological traits and appearance of intracellular vesiculation due to OMV intake.

In order to assess potential cytotoxicity of *Pg*-OMVs towards macrophages, the CCK-8 assay was performed and the overall cell viability assessed. Figure 1g, which shows a plot with the results of this analysis revealed the evolution in overall cell viability at 24 h incubation, revealed that the maximum tolerated treatment consisted of a dose of 0.25 μg/mL *Pg*-OMVs with 0.02 μg/mL of IFN-*γ*. This treatment gave a statistically similar result to a treatment with a dose of 0.10 μg/mL LPS with 0.02 μg/mL of IFN-*γ*. Addition to the culture of larger amounts of *Pg*-OMVs resulted in a progressive cell-viability reduction with a low saturation threshold at concentrations ≥1.25 μg/mL.

**Figure 2:**
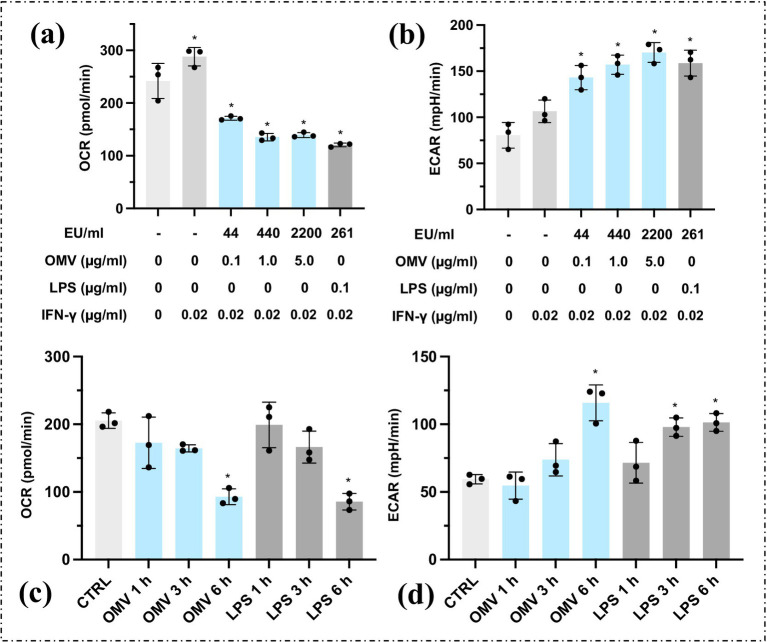
OMV-induced metabolic adaptations in RAW264.7 cells; OCR **(a)** and ECAR **(b)** plots after exposure to increasing concentrations of *Pg*-OMVs or LPS in combination with IFN-*γ* for 6 h. In **(c,d)**, OCR and ECAR plots for cells treated with *Pg*-OMVs (1.0 μg/mL) or LPS together with IFN-γ as a function of time. *Pg*-OMVs suppressed mitochondrial respiration and enhanced glycolysis in a dose- and time-dependent manner, indicating macrophage polarization toward a pro-inflammatory phenotype. Data are presented as means ± SD from three independent experiments. Statistical significance was assessed by one-way ANOVA followed by Dunnett’s multiple-comparison test with the control group as a reference. *p < 0.05.

The effect of *Pg*-OMVs or LPS on macrophage culture was also recorded by means of analyses of cellular bioenergetics (Figure 2). RAW 264.7 cells were treated with varying concentrations of OMVs or LPS in the presence of IFN-γ, and the activities of the two major bioenergetic pathways, mitochondrial respiration and glycolysis, were assessed using extracellular flux analysis. These further assessments characterize the impact of *Pg*-OMVs on metabolic adaptations associated with inflammation and enable to determine whether *Pg*-OMVs are capable of eliciting macrophage activation. These additional analyses were based on OCR (cf. Figures 2a,c as a function of OMV concentration and time, respectively) and ECAR (cf. Figures 2b,d as a function of OMV concentration and time, respectively) assays. After 6 h exposure to OMVs, OCR, as a parameter representative of mitochondrial aerobic respiration, was markedly reduced in a concentration-dependent manner (Figure 2a). Accordingly, OMV treatment resulted in an increase in glycolytic activity, as reflected by an elevated ECAR (Figure 2b). This trend reveals how *Pg*-OMVs suppressed mitochondrial respiration and enhanced glycolysis in RAW264.7 cells in a dose-dependent manner, a metabolic shift being indicative of macrophage polarization toward a pro-inflammatory phenotype. The magnitude of the OMV-induced metabolic shift toward glycolysis was comparable to that induced by LPS plus IFN-γ, a conventional stimulus commonly used to drive macrophage polarization toward the pro-inflammatory phenotype. The measured endotoxin activities were within a comparable range between *Pg*-OMVs and purified LPS (440 EU/mL for 1 μg/mL Pg-OMV and 261 EU/mL for 0.1 μg/mL LPS), suggesting that OMV-associated LPS is the primary effector underlying the observed metabolic reprogramming. Based on these results, a concentration of 1 μg/mL OMV was selected for all subsequent experiments. Next, RAW264.7 cells were treated with OMVs for 1, 3, or 6 h to assess time-dependent effects on cellular metabolism. OMV treatment resulted in a time-dependent reduction in OCR and a concomitant increase in ECAR, with statistical significance observed after 6 h of exposure (Figures 2c,d). Thus, *Pg*-OMVs potently induced metabolic reprogramming in RAW264.7 cells, indicating that bacterial components within OMVs can trigger macrophage activation in the absence of intact bacteria. As a consequence of cellular activation, a metabolic reprogramming is expected, which enables the cells to meet their high energetic and biosynthetic demands, thereby supporting their pro-inflammatory functions.

**Figure 3:**
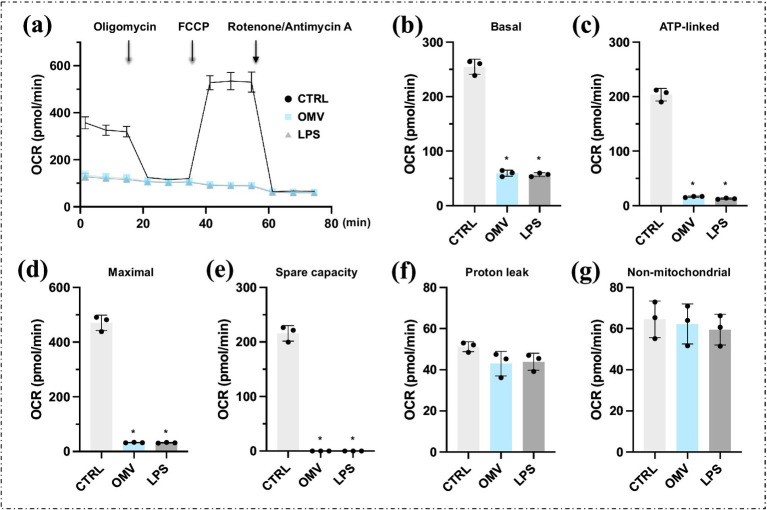
Measurement of key parameters of mitochondrial function in RAW264.7 cells; **(a)** Assessments of mitochondrial respiration by the seahorse XF Cell Mito Stress Test for cells treated with *Pg*-OMVs or LPS in combination with IFN-γ for 6 h. The test evaluates multiple components of mitochondrial function, including **(b)** basal respiration, **(c)** ATP-linked respiration, **(d)** maximal respiration, **(e)** spare respiratory capacity, **(f)** proton leak, and **(g)** non-mitochondrial oxygen consumption. OCR was monitored following sequential injections of oligomycin (ATP synthase inhibitor), FCCP (uncoupler), and a mixture of rotenone and antimycin A (complex I and III inhibitors). Data are presented as means ± SD from three independent experiments. Statistical significance was assessed by one-way ANOVA followed by Dunnett’s multiple-comparison test with the control group as a reference. *p < 0.05.

To further characterize the OMV-induced metabolic adaptations in macrophages, we utilized the Cell Mito Stress Test, which measures selected key parameters of aerobic respiration and provides a comprehensive profile of mitochondrial function (Figure 3a). OMV treatment almost completely suppressed mitochondrial respiration required for energy production (Figures 3b,c). Furthermore, the addition of the uncoupler FCCP failed to elicit any increase in OCR, indicating an essentially complete loss of mitochondrial spare respiratory capacity (Figures 3d,e). Despite the profound inhibition of oxygen consumption supporting energy production in mitochondria, OMV treatment did not alter proton leak–associated respiration (Figure 3f) nor non-mitochondrial oxygen consumption (Figure 3g). These results indicate that OMVs induce a metabolic phenotype closely resembling that of classically activated (LPS + IFN-*γ*–stimulated) inflammatory macrophages.

**Figure 4:**
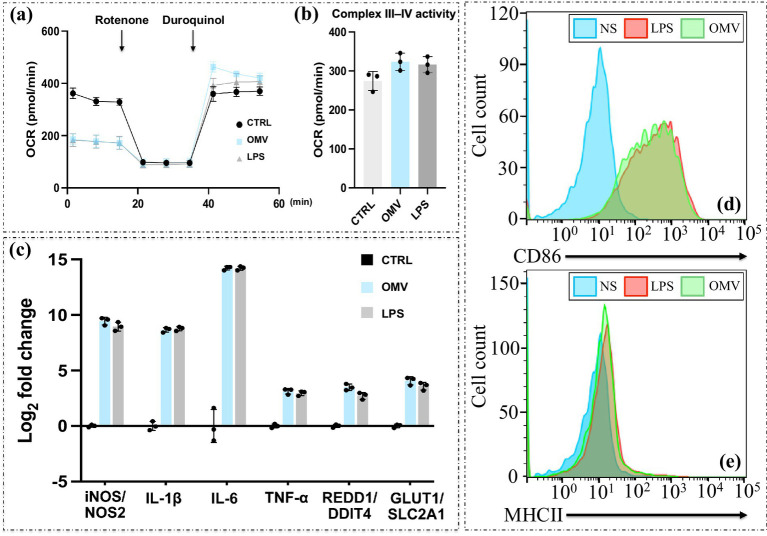
Preserved mitochondrial respiratory activity in OMV-treated cells: **(a)** OCR results obtained by treating RAW264.7 cells with *Pg*-OMVs or LPS in combination with IFN-γ for 6 h. Cells were treated with rotenone to inhibit complex I, followed by the addition of duroquinol, which donates electrons directly to complex III. The change in OCR in response to duroquinol was monitored to assess complex III–IV activity. No significant difference in duroquinol-supported oxygen consumption was observed between control and *Pg*-OMVs-stimulated cells **(b)**, indicating that complex III–IV–mediated respiration remains intact despite *Pg*-OMV-induced suppression of basal mitochondrial respiration. Data are presented as means ± SD from three independent experiments. Statistical significance was assessed by one-way ANOVA followed by Dunnett’s multiple-comparison test with the control group as a reference. **(c)** A gene expression analysis in OMV-activated macrophages is offered; results of the expression levels for selected inflammation-related genes as determined by qPCR. RAW264.7 cells were stimulated with *Pg*-OMVs or LPS in combination with IFN-γ for 16 h. Data are shown as log_2_ fold changes relative to untreated control cells. All analyzed genes (cf. labels in inset) showed statistically significant differences compared with the control in both OMV- and LPS-stimulated groups (*p* < 0.05). Finally, **(d,e)** representative histograms are given, which show CD86 and MHC-II staining intensity, respectively, in non-stimulated, LPS-stimulated, and *Pg*-OMV-stimulated RAW264.7 cells (cf. labels in inset).

To distinguish OMV-induced inflammatory metabolic switch from nonspecific mitochondrial toxicity, we next assessed the integrity of the electron transport chain upon exposure to *Pg*-OMVs. Electron flow through Complex I was blocked with rotenone, thereby preventing upstream electron input into the respiratory chain. Duroquinol was subsequently applied to directly donate electrons to Complex III (Figure 4a). In OMV-treated cells, duroquinol restored OCR to levels comparable to those observed in control cells, demonstrating that complexes III–IV remained fully functional (Figure 4b). The OMV–driven decrease in mitochondrial respiration was not due to mitochondrial damage induced by virulence factors contained within OMVs, but rather reflects regulated metabolic remodeling.

In order to assess how *Pg-*OMVs trigger upregulation of inflammatory markers in macrophages, we next examined whether OMVs modulate the expression of inflammatory genes in macrophages. Treatment with OMVs resulted in a significant upregulation of pro-inflammatory cytokines (IL-1*β*, IL-6, and TNF-*α*), inducible nitric oxide synthase (iNOS/NOS2), and genes associated with glycolytic activation (Redd1/Ddit4 and Glut1/Slc2a1) comparable to the effects observed with LPS + IFN-*γ* stimulation (Figure 4c).

It is known that macrophages play an important role in regulating both innate and adaptive immune response. When stimutated by LPS or other pathogen-associated molecular patterns (e.g., *Pg*-OMVs, in the present experiments), macrophages become activated, leading to increased expression of cell surface markers such as co-stimulatory molecules and major histocompatibility complex II. These molecules are essential for cell activation or immune responses, therefore, examinig the expression level of these cell surface markers is appropriate for determining the state of macrophages. In order to determine which proteins are expressed on the surface of macrophages, we stimulated macrophages with *Pg*-OMV/IFN-γ and examined the expression of CD86, co-stimulatory molecules, and MHC II by means of flow cytometry. The results of these characterizations, which are given in Figures 4d,e, respectively, show that the expression levels of CD86 and MHC II in *Pg*-OMV/IFN-γ stimulated RAW 264.7 cells systematically increased as compared to unstimulated cells. MHC II positive subset in RAW 264.7 was 29.1% (no stimulation), 60.5% (LPS-stimulated), 55.1% (Pg-OMV-stimulated), respectively. CD86 contains two domains, an immunogloblin variable-like domain (IgV) and an immunoglobulin constant-like domain (IgC), both belonging to the immunoglobulin superfamily. MHC II is a transmembrane glysoprotein composed of α-chain and β-chain. Both chains span the cell membrane and form a peptide-binding site on the cell surface. An important finding in the flow cytometry context was that *Pg-*OMVs induced an activation profile almost the same as that elicited by LPS. Collectively, these findings demonstrate that OMVs function as immunomodulatory particles capable of driving macrophages toward a pro-inflammatory phenotype.

**Figure 5:**
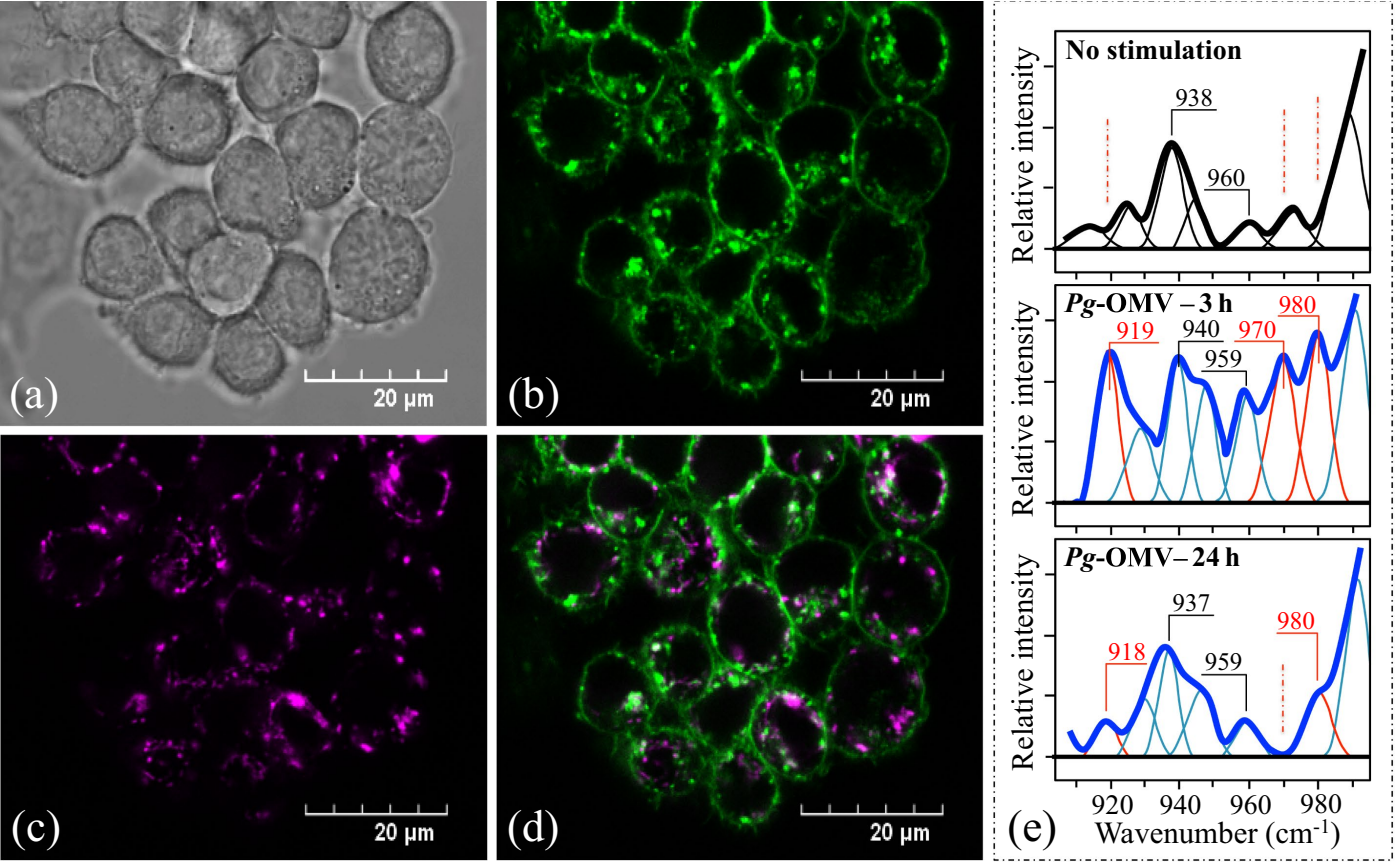
Confocal imaging of *Pg*-OMV internalization in RAW264.7 cells; RAW264.7 cells were incubated with fluorescently labeled *Pg*-OMVs (1 μg/mL) for 3 h. The plasma membrane is shown in green and *Pg*-OMVs in magenta: **(a)** Bright-field image, **(b)** plasma membrane, **(c)** *Pg*-OMVs, and **(d)** merged image of **(b,c)**. Representative images from three independent experiments are shown. In **(e)**, high-resolution Raman spectra in the wavenumber interval 910–990 cm^−1^ are compared for unexposed and for *Pg*-OMV-stimulated RAW264.7 cells after 3 and 24 h (from top to bottom; cf. labels in inset), in order to clarify the integrity of the OMVs after cell penetration.

Finally, in order to confirm the interaction between *Pg*-OMVs and target cells, fluorescently labeled *Pg*-OMVs were incubated with RAW264.7 cells and visualized by confocal microscopy. After 3 h of incubation, confocal imaging detected a number of punctate fluorescent signals within the cytoplasmic compartment (Figures 5a–d; cf. description in the caption). These results support the hypothesis of partial cell internalization of *Pg*-OMVs, suggesting that *Pg*-OMVs exert their effects on cells through direct interaction and subsequent uptake. Figure 5e shows high-resolution Raman spectra collected in the wavenumber interval 910 ~ 990 cm^−1^ from unexposed and *Pg*-OMV-stimulated RAW264.7 cells, in order to clarify the integrity of the OMVs structure after cell penetration. The significant spectral differences recorded will be discussed in detail in the next section.

### 3.2 Raman spectroscopy assessments

**Figure 6:**
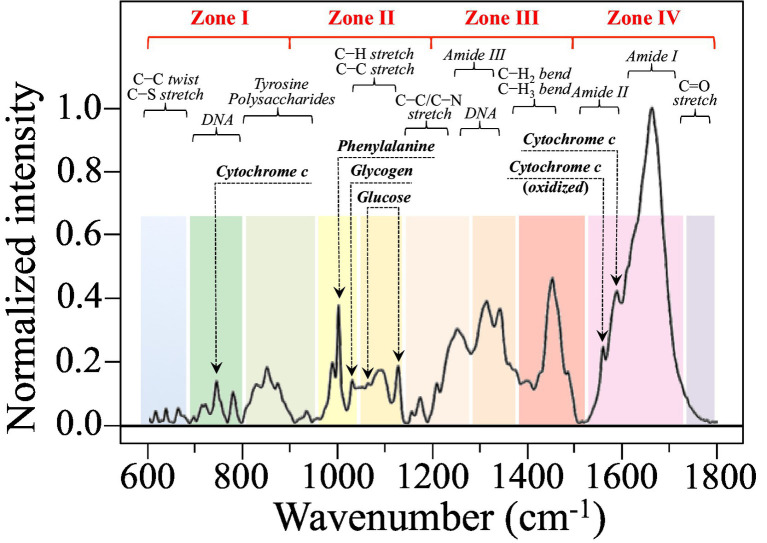
Normalized Raman spectrum of RAW264.7 cells cultured for 6 h without any immunostimulation; the spectrum is divided into zones I–IV for increasing wavenumbers and key vibrational modes and molecules are labeled in inset.

**Figure 7:**
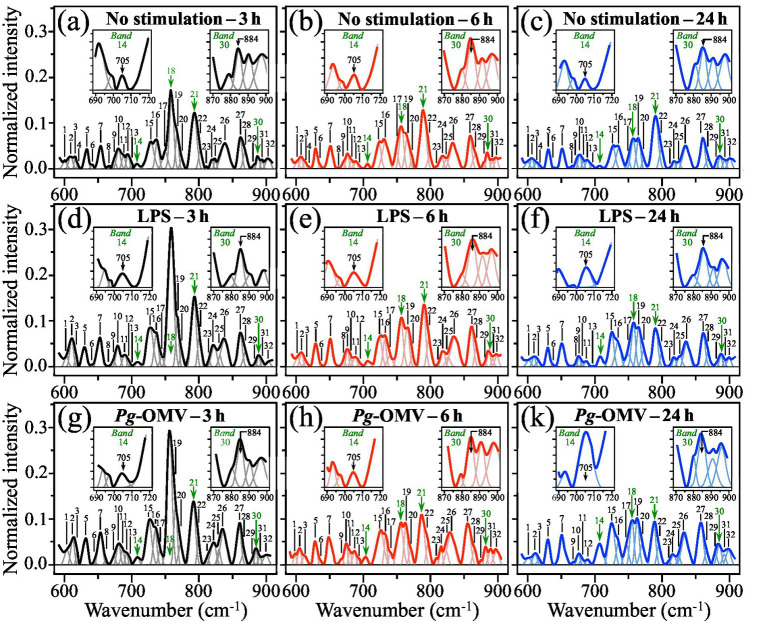
High spectrally resolved Raman spectra collected at increasing times (3, 6, and 24 h) in Zone I (600–900 cm^−1^) on RAW264.7 cells with no stimulation **(a–c)**, stimulated with LPS **(d–f)**, and stimulated with *Pg*-OMVs **(g–k)**. Spectra were deconvoluted and all sub-bands numbered (cf. vibrational origins in Table S-III); in inset, the spectral zones of lipid-related Bands 14 and 30 are enlarged and given in inset to each figure. Key signals used in Raman analyses are emphasized with arrows in green color.

**Figure 8:**
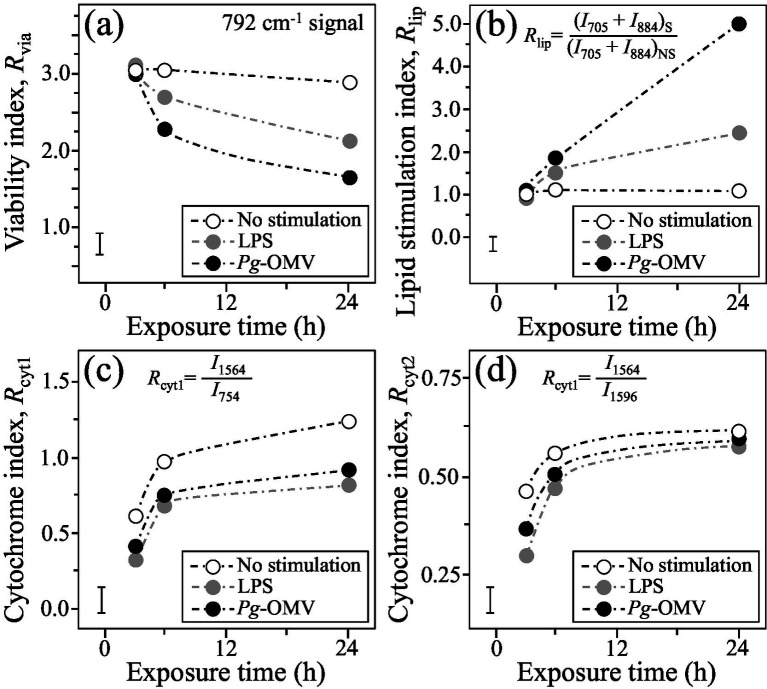
Plots of **(a)** viability index, *R*_via_, **(b)** lipid stimulation index, *R*_lip_, and cytochrome indexes, **(c)** *R*_cyt1_, and **(d)** *R*_cyt2_, as retrieved from areal intensities of selected Raman signals in the spectra of RAW264.7 cells with no stimulation, stimulated with LPS, and stimulated with *Pg*-OMVs (cf. labels in inset).

Figure 6 shows a normalized Raman spectrum of pre-cultured and 24 h-supplemented RAW264.7 macrophages. The spectrum was divided into 4 different spectral zones (referred to as Zones I ~ IV; cf. labels in inset), including vibrational characteristics related to main molecules related to the following discussion. Based on the Raman response of the control macrophage culture, we proceeded with the Raman characterization of macrophages under different stimulation conditions. Figure 7 shows Zone I for the high spectrally resolved spectra of living macrophage cultures after 3, 6, and 24 h for no stimulation, LPS stimulation, and *Pg*-OMVs stimulation (1 μg/mL; cf. (a)/(b)/(c), (d)/(e)/(f), and (g)/(h)/(k), respectively). All spectra in this zone showed strong similarity, but also clearly different details, which are traceable to fundamental physiological changes in the stimulated cells. All deconvoluted band components in the spectrum of untreated RAW264.7 macrophages were numbered and their proposed vibrational origins indicated in the Supplementary Table S3. The relatively strong signal at ~792 cm^−1^ (i.e., Band 21 in Figure 7), which is representative of nucleic acid (pyrimidine ring-breathing and phosphodiester backbone mode) (Notingher et al., 2004a) showed clear trends with both time and stimulation (cf. relative areal intensity plots in Figure 8a), in agreement with that measured by conventional viability test (cf. Figure 1g). The 792 cm^−1^ Raman band is a well-established nucleic-acid marker, which is expected to decrease when DNA becomes condensed, oxidized, fragmented, or transcriptionally less active, or when nucleic-acid content per cell declines (Notingher et al., 2004b). Because the above-mentioned vibrational modes depend on the integrity and accessibility of DNA, reductions in the 792 cm^−1^ band could also be assumed as an indicator of chromatin condensation, reduced transcriptional activity, DNA damage, or early apoptosis, more generally reflecting a loss of well-ordered pyrimidine-rich DNA structure within cells. In the present context, being a decrease in the 792 cm^−1^ signal fully consistent with a lower DNA metabolic activity, we defined a Raman viability index, *R*_via_, simply as the relative areal intensity of this DNA-related band and interpreted it as a marker for macrophage shifting towards an immune-evasion configuration. Plots in Figure 8a show that stimulation by *Pg*-OMVs created the most severe environment for macrophages’ viability.

**Figure 9:**
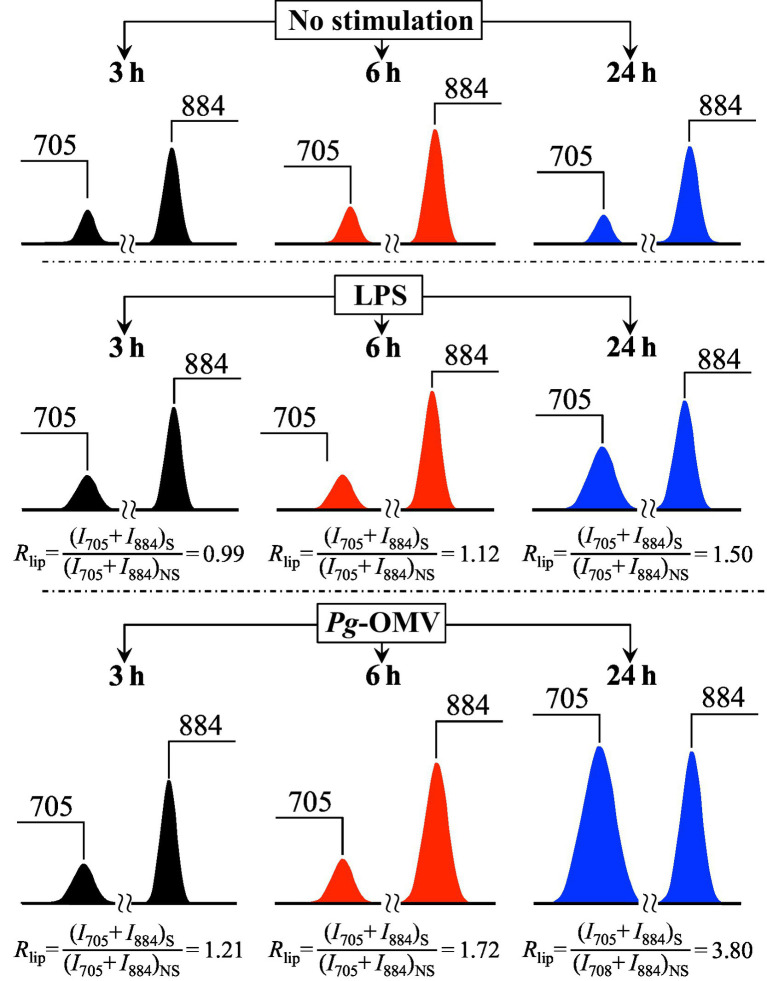
Key lipid-related bands as extracted from time-resolved and deconvoluted Raman spectra of RAW264.7 cells with no stimulation, stimulated with LPS, and stimulated with *Pg*-OMVs (cf. labels in inset). In inset, the values of lipid stimulation index, *R*_lip_, are given as computed from the respective areal fractions.

Another key-signal in Zone I, which underwent morphological variations as a function of stimulation/time, was the Raman band at ~705 cm^−1^ (cf. Band 14 in Figure 7), a wavenumber commonly assigned to cholesterol/sterol vibrations (i.e., out-of-plane bending of C–H bonds in sterol ring) (Czamara et al., 2015). However, the 705 cm^−1^ band also represents a signal of medium intensity belonging to sphingomyelin (i.e., N^+^(CH_3_)_3_ symmetric stretching in the phosphocholine group) (Czamara et al., 2015). An additional medium-weak band at 884 cm^−1^, which could be assigned to N^+^(CH_3_)_3_ asymmetric stretching in the phosphodiester backbone (while also corresponding to C–C stretching in the sterol ring of cholesterol) (Czamara et al., 2015), pairs with the 705 cm^−1^ band in characterizing the lipid chemistry in living macrophage cultures. Enlarged spectral zones for both bands are given in inset to Figure 7 for better visualization. It should be noted that, despite not being the bands with the strongest intensity in lipids, the two signals at 705 and 884 cm^−1^ are the most suitable signals for screening lipid metabolism, because they can unequivocally be assigned to lipids without overlaps by other molecules (cf. Table S-III, which displays assignments for all deconvoluted bands of Zone I). Accordingly, we defined a spectroscopic parameter referred to as lipid stimulation index, *R*_lip_ = (*I*_705_ + *I*_884_)_S_/(*I*_705_ + *I*_884_)_NS_, which gives the ratio between the cumulative (areal) intensity of the 705 and 884 cm^−1^ signals under stimulated and non-stimulated conditions (cf. subscripts S and NS, respectively). The computed *R*_lip_ values are plotted as a function of time in Figure 8b after extracting them from the respective spectra; Figure 9 shows the relative intensities of lipid bands extracted from spectra collected on non-stimulated, LPS-, or *Pg*-OMV-stimulated living macrophage cultures. Significant increases for the lipid marker, *R*_lip_, were only observed after 24 h in both cases of *Pg*-OMVs and LPS stimulation, the former being again clearly more marked than the latter. A delayed increase in lipid metabolism is consistent with OMV-driven metabolic and lipid-handling reprogramming that promotes cholesterol esterification and lipid-droplet biogenesis. Macrophages recognize the pathogen-associated molecular patterns (e.g., LPS), thus activating inflammatory pathways and transcriptional programs that increase lipid uptake and esterification, with nascent lipid droplets becoming spectrally detectable only after several hours. More specifically, the time-dependent increase of the Raman feature at ~884 cm^−1^ in OMV-treated macrophages is consistent with accumulation of neutral lipids and lipid-droplet biogenesis, namely, cellular cholesterol-ester and triglyceride levels, which paralleled in the spectral changes.

**Figure 10:**
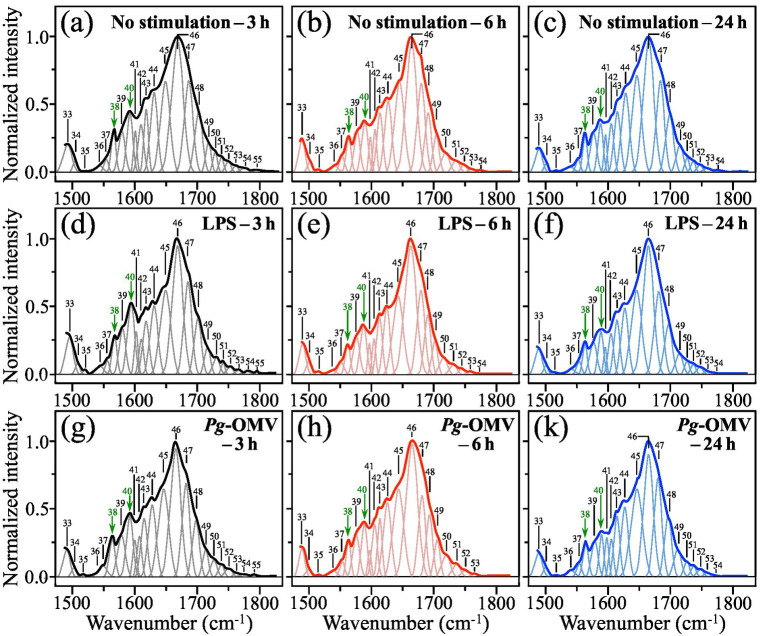
High spectrally resolved Raman spectra collected at increasing times (3, 6, and 24 h) in Zone IV (1500–1800 cm^−1^) on RAW264.7 cells with no stimulation **(a–c)**, stimulated with LPS **(d–f)**, and stimulated with *Pg*-OMVs **(g–k)**. Spectra were deconvoluted and all sub-bands numbered (cf. vibrational origins in Table S-IV); Bands 38 and 40, which were analyzed together with Band 18 (in Zone I) to screen cytochrome *c* oxidation are emphasized with arrows in green color.

**Figure 11:**
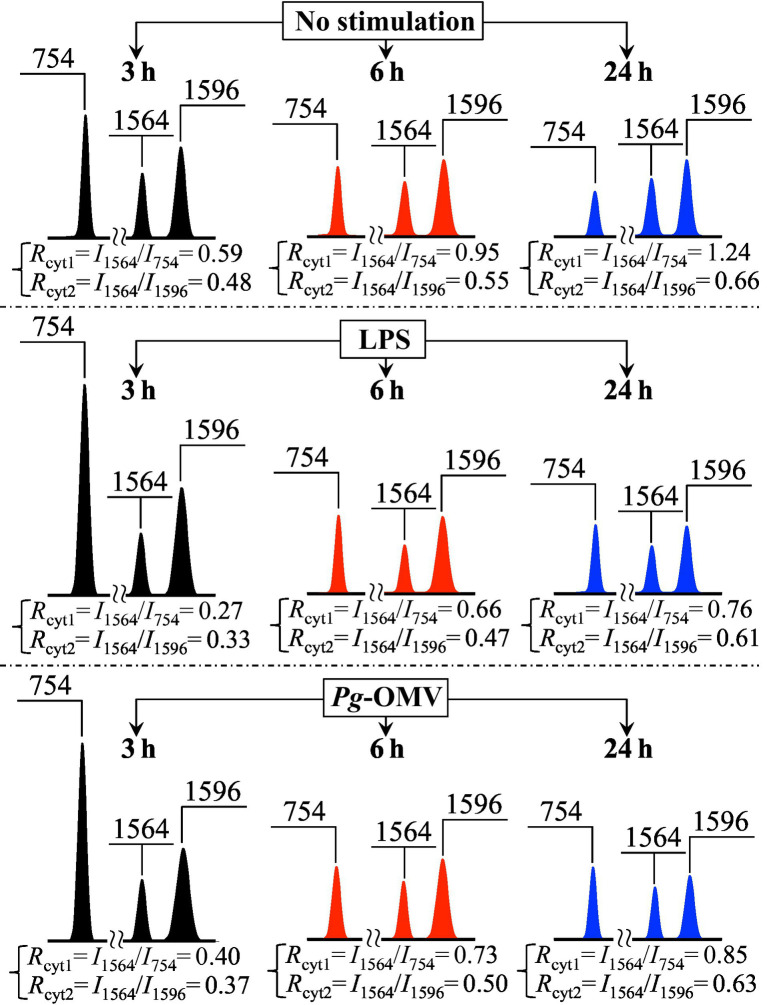
Key cytochrome *c*-related bands as extracted from time-resolved and deconvoluted Raman spectra of RAW264.7 cells with no stimulation, stimulated with LPS, and stimulated with *Pg*-OMVs (cf. labels in inset). In inset, the values of cytochrome indexes, *R*_cyt1_ and R_cyt2_ are given as computed from the respective areal fractions.

An additional Raman marker in Zone I is represented by its most prominent band located at 754 cm^−1^ (cf. Band 18 in Figure 7), which is assigned to the pyrrole breathing mode of cytochrome *c* (Hu et al., 1993). This band showed a decreasing trend in any of the tested conditions, but such trend was clearly more marked in the case of stimulation by both LPS and *Pg*-OMVs. In order to obtain a complete Raman analysis of mitochondrial oxidation, however, it is necessary to monitor additional signals from cytochrome *c* located in Zone IV, namely, at 1564 and 1,596 cm^−1^ (both contributed by C=C stretching but in different bonds of the porphyrin ring of the heme molecule, and C–C/C–N stretching in different extents) (Hu et al., 1993; Spiro, 1985; Okada et al., 2012). These two bands both display in the Zone IV spectra given in Figure 10 (cf. Bands 38 and 40, respectively; assignments for all deconvoluted bands of Zone IV are given in the Supplementary Table S4). Cytochrome *c* is a redox-active heme protein, which is released by the mitochondria into the cytosol during apoptosis and undergoes redox state changes from Fe^2+^ to Fe^3+^ and vice versa (Brown and Borutaite, 2008). The method of using Raman assessments to follow *in situ* the fate of individual cells was pioneered by Puppels et al. (1990, 1993), Uzunbajakava et al. (2003), and Kitt et al. (2017). Cytochrome *c* is a very sensitive Raman biomarker, its interconvertible switch from reduced (Fe^2+^) to oxidized (Fe^3+^) heme being marked by distinct signals. An important peculiarity in assessing cytochrome reduction/oxidation is that its Raman signals are greatly enhanced with respect to overlapping signals from other proteins when using a 532 nm (green) laser irradiation, due to resonance Raman conditions (i.e., which is the case here). According to Okada et al. (2012), the signals at 754 and 1,596 cm^−1^ are characteristic of both reduced and oxidized cytochrome *c*, while the signal at 1564 cm^−1^ is strongly enhanced only in the oxidized state. Accordingly, we defined two distinct Raman cytochrome indexes, as follows: *R*_cyt1_ = *I*_1564_/*I*_754_ and *R*_cyt2_ = *I*_1564_/*I*_1596_. Both indexes increased with increasing the oxidation extents of the heme protein in the macrophage cells. Figure 11 shows the selected cytochrome *c* signals, (i.e., Bands 18, 38, and 40), after extraction from the Raman spectra of non-stimulated, LPS-, and *Pg*-OMV-stimulated cell cultures given in Figures 7, 10. In inset, both *R*_cyt1_ and *R*_cyt2_ indexes are given, as computed from the respective areal ratios. Values for both indexes are finally plotted as a function of time in Figures 8c,d in order to compare macrophages under different stress conditions. The Raman analysis of the cytochrome *c* redox bands reveals that macrophages exposed to either LPS or *Pg*-OMVs displayed a *less oxidized* mitochondrial state as compared to control macrophages cultured under identical conditions without any stimulation. Although mitochondrial oxidation increased over time in all testing conditions (and consistently according to both *R*_cyt1_ and *R*_cyt2_ indexes), the extent of oxidation was markedly attenuated in the case of both LPS and OMVs stimulations. This observation suggests that *Pg*-OMVs partially *inhibit* mitochondrial electron transport activity, likely through the combined action of lipopolysaccharides, lipoproteins, and gingipains that alter mitochondrial integrity and cellular metabolism. Such impairment is consistent with a metabolic reprogramming of macrophages toward a more glycolytic, pro-inflammatory phenotype, characterized by reduced oxidative phosphorylation and increased reductive stress. In other words, the decrease in cytochrome *c* oxidation reflects a shift in energy metabolism and redox balance that contributes to *Pg* immune evasion through a strategy that limits efficient mitochondrial activation during macrophage response.

**Figure 12:**
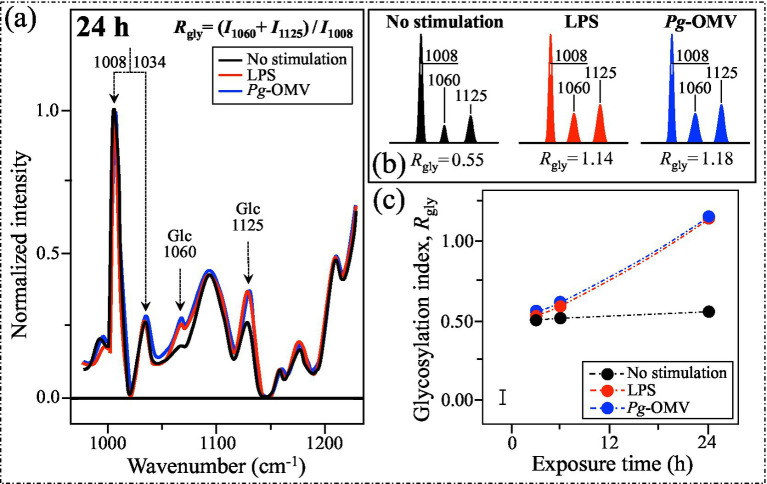
**(a)** High spectrally resolved Raman spectra collected at 24 h in the wavenumber interval 950–1,250 cm^−1^ of Zone II on RAW264.7 cells with no stimulation, stimulated with LPS, and stimulated with *Pg*-OMVs. Two key-signals at 1060 and 1,125 cm^−1^ (labeled as Glc) were deconvoluted and assumed as markers for glycolysis enhancements. In **(b)**, these bands are deconvoluted, extracted, and compared with the phenylalanine bands at 1008 cm^−1^, which remained unchanged upon stimulation. The Raman glycosylation index, *R*_gly_, was computed from areal intensities and plotted in **(c)** as a function of time and type of stimulation.

In order to substantiate the above interpretation, we further monitored Raman bands related to the glycolytic activity of the cell culture. The two bands at 1060 and 1,125 cm^−1^ (both assigned to C–O stretching in the primary alcohol group coupled with C–C stretching in the pyranose ring) (Wierchigroch et al., 2017) can be assumed as markers for glycolysis enhancements (Shao et al., 2012). Figure 12a shows in overlap Raman spectra in the wavenumber interval 950 ~ 1,250 cm^−1^ (within Zone II) after 24 h culture of non-stimulated, LPS-, and Pg-OMV-stimulated cells. As seen, for the same intensity of the phenylalanine bands at 1008 and 1,035 cm^−1^ (breathing and in-plane C–H deformation in the phenyl ring, respectively) (Hernandez et al., 2013; Zhu et al., 2011), the two markers of glycolytic activity (i.e., the signals at 1060 and 1,125 cm^−1^, labeled as Glc) appeared similarly enhanced in both types of cellular stimulation. Accordingly, a Raman glycosylation index could be defined from the (areal) intensity ratio of the above bands, as follows: *R*_gly_ = (*I*_1060_ + *I*_1125_)/*I*_1008_. Deconvoluted bands are plotted and compared in Figure 12b for different cell-culture conditions, and the respective values of glycosylation indexes, *R*_gly_, are shown in inset. In Figure 12c, a plot is given of the *R*_gly_ index as a function of time for both LPS- and *Pg*-OMV-stimulated cells, in comparison with non-stimulated control cells. The observed trend suggests that *Pg*-OMV-associated virulence factors, including LPS and gingipains, drive a pronounced pro-inflammatory activation that enhances GLUT1-mediated glucose uptake and induces a Warburg-like metabolic reprogramming toward aerobic glycolysis. This metabolic reaction results in an enlarged intracellular pool of glucose and its intermediates. In parallel, inflammatory signaling promotes glycogen synthesis and storage, contributing additional carbohydrate-derived Raman features with spectral profiles similar to glucose. The increased glucose-like signals could also partly derive from the internalization of *Pg*-OMVs themselves, which contain glycoproteins, glycolipids, and other sugar-rich components. Moreover, the oxidative and inflammatory stress elicited by *Pg*-OMVs redirects part of glucose flux into the pentose phosphate and hexosamine biosynthesis pathways, further expanding intracellular carbohydrate reservoirs. Together, these processes account for the heightened Raman intensities of pyranose-ring vibrational modes observed here. Finally, the late (i.e., at 24 h; cf. Figure 12c) increase in the pyranose-ring bands likely reflects metabolic and structural adaptation of macrophages to *Pg*-OMVs, following the earlier transcriptional changes leading to the observed 792 cm^−1^ DNA-band decrease (cf. Figure 8a). Such delay is consistent with the time required for metabolic reprogramming and accumulation of detectable metabolite levels.

A final but important detail resided in searching Raman spectra for hints on whether or not OMVs conserved their structural integrity after becoming incorporated into RAW264.7 macrophages. Since the above spectroscopic interpretations entirely rely on the effects of the OMVs’ composite molecular cargo on the cells’ physiological response, this distinction is indeed critical. In this specific issue, the high spectrally resolved Raman spectra in the wavenumber interval 910 ~ 990 cm^−1^ offer a chance to screen Raman signals only belonging to OMVs and not to cells. Spectral comparisons between unexposed and *Pg*-OMV-stimulated RAW264.7 cells after 3 and 24 h (cf. Figure 5e and labels in inset) revealed several significant differences, as follows: (i) three relatively strong signals at 919, 970 and 980 cm^−1^ were recorded in *Pg*-OMV-stimulated cells after 3 h, which were not present in unexposed cells; (ii) the above three signals were significantly weakened or even disappeared (i.e., in the case of the band at 970 cm^−1^) after OMVs exposure for 24 h; (iii) a relatively strong signal at 937 ~ 940 cm^−1^ stemmed as relatively unchanged across different cell-culture conditions; and, (iv) a signal at ~960 cm^−1^, relatively weak in non-stimulated cells, reached a more than twice higher relative intensity after 3 h since OMVs’ exposure, but it returned to low intensity levels after 24 h exposure.

The doublet at 919 and 970 cm^−1^ can be ascribed S–O stretching in oxygen-containing inorganosulfur compounds (Sato et al., 1985), which previous Raman studies identified among other toxins comprised within *Pg*-OMVs (Pezzotti et al., 2023). On the other hand, the band centered at 980 cm^−1^ could be assigned to –PO_3_^2−^ stretching in phosphoglycerol dihydroceramide, namely the main molecular component of the *Pg*-OMVs’ membrane. The significant intensity reduction or even disappearance of these three spectral bands after 24 h can thus be interpreted as a spectroscopic proof for a loss of integrity in *Pg*-OMVs after incorporation in the macrophages’ cytoplasm. The validity of this hypothesis is backed by the initial intensity increase at 3 h of the Raman doublet at 959 cm^−1^ and its successive decrease after 24 h to return to approximately the same level of the unexposed sample. As a matter of fact, the 959 cm^−1^ band can be assigned to a stretching mode in the phosphate groups of both phospholipids and LPS present in *Pg*-OMVs (Pezzotti et al., 2023). Once the OMVs’ membrane is enzymatically or oxidatively disrupted, internal toxins could gradually be degraded, so their signals tend to disappear after 24 h. Finally, the conspicuous invariance of the band at ~938 cm^−1^ with both OMV-exposure time could be ascribed to the fact that this band represents C–C stretching in several different molecules, including amino acids and proteins (Zhu et al., 2011); therefore, it results to be less affected by the presence/absence of *Pg*-OMVs.

In summary, Raman data contained information fully compatible with that retrieved from immunological assays and indicating how *Pg*-OMVs induced a partial inhibition of mitochondrial oxidation and, concurrently, a shift toward glycolytic, pro-inflammatory macrophage metabolism, consistent with immune activation and bacterial immune evasion strategies. As a further proof of the depth of the obtained Raman information, the presented spectroscopic analyses were also quantitative in showing, consistently with immunological tests, a stronger impact of *Pg*-OMVs as compared to LPS only (cf. Figure 8).

## 4 Discussion

### 4.1 Comparing Raman markers with results of immunological assays

According to literature, a mechanistic chain of events could be foreseen, which is consistently supported by both Raman and biological tests performed in this study, as follows (Fleetwood et al., 2017; Wall et al., 2022; Goicoechea et al., 2023; Sartorio et al., 2022; Jiang and Huang, 2024; Qi et al., 2003; Birbes et al., 2005; Novgorodov et al., 2019):

(i) OMV components (LPS, gingipains, bacterial sphingolipids) inhibit the electron transport chain; (ii) cytochrome *c* becomes more reduced and oxidative phosphorylation falls; (iii) metabolic reprogramming, including an enhanced glycolysis (cf. intensity increase in bands at 1060 and 1,125 cm^−1^ in the presence of both LPS and OMVs), takes place and adds upon altered endoplasmic reticulum–mitochondria sterol trafficking and stress signalling; and (iv) activation/misregulation of cholesterol biosynthetic/trafficking pathways leads to intracellular cholesterol accumulation and formation of foam droplets. In parallel, OMV-delivered sphingolipids and OMV-driven changes in sphingomyelinase/synthase activities increase cellular sphingomyelin content; mitochondrial sphingolipid changes further affect cytochrome *c* dynamics, closing a feedback loop.

Regarding the above points (i) ~ (iii), IFNγ is a potent activator of macrophage respiration and typically enhances mitochondrial oxidative metabolism to support antimicrobial functions, leading to an increase in OCR. However, according to Figure 2, when *Pg*-OMVs are added concurrently with IFNγ, this respiratory stimulation is counteracted because *Pg*-OMVs deliver bacterial toxins and virulence factors that impair electron transport, particularly at complexes I and IV. Since OCR is a direct readout of mitochondrial electron transport, its lower values reflect a more reduced cytochrome *c*. In other words, while IFNγ alone promotes oxidative metabolism, *Pg*-OMVs impose a dominant inhibitory effect on the electron transport chain, shifting the metabolic balance back toward glycolysis and lowering OCR despite the presence of IFNγ. The concurrent increase in ECAR, (cf. Figure 2) is an indicator of metabolic shift from mitochondrial respiration to glycolysis, since it refers to acidification of the medium, as mainly due to lactate production from glycolysis.

Remarkably, Raman spectroscopy recorded a scenario fully consistent with both the oxidative trend of cytochrome *c* and the level of glycolysis, and provided their quantitative assessments through the parameters *R*_cyt1_ and *R*_cyt2_ (cf. Figures 8c,d, respectively), and *R*_gly_ (cf. Figure 12c), respectively. Macrophage reaction to the sequence of injections in Cell Mito Stress Test (cf. Figure 3) further revealed that *Pg*-OMVs profoundly blunted the dynamic mitochondrial responses normally observed during the Cell Mito Stress Test (cf. control sample in Figure 3a), producing a flattened and only mildly decreasing OCR trace. This occurs because *Pg*-OMVs contain bacterial toxins and virulence factors that inhibit electron transport, thereby suppressing basal respiration and limiting the ability of mitochondria to respond to pharmacological challenges. As a result, oligomycin produces no additional drop in OCR (ATP-linked respiration is already minimal), FCCP fails to induce a maximal respiratory burst (spare respiratory capacity is lost), and rotenone/antimycin A produce only a small further decrease. In other words, *Pg*-OMVs place mitochondria into a functionally constrained, low-capacity state in which all stress-test injections elicit minimal changes, generating a monotonic, flattened time-lapse profile characteristic of globally impaired oxidative phosphorylation. Repeating the time-lapse assay with rotenone and duroquinol revealed that *Pg*-OMVs selectively impaired electron entry through Complex I. Rotenone, a Complex I inhibitor, flattened the OCR curve in *Pg*-OMV–treated macrophages far more than in controls, indicating that Pg-OMVs had already suppressed endogenous Complex I activity and left little remaining respiration for rotenone to inhibit. In contrast, duroquinol, which is an artificial electron donor that bypasses Complex I and directly feeds electrons into Complex III, restored OCR to levels comparable to or even slightly higher than control cells. This rescue effect demonstrates that downstream components of the electron transport chain remained functional and that the principal deficit induced by *Pg*-OMVs lies specifically at or upstream of Complex I. Taken together, these findings show that *Pg*-OMVs impose a targeted blockade of Complex I–dependent respiration, which can be overcome when electrons are supplied downstream of the inhibited step. *Pg*-OMVs also induced a robust inflammatory transcriptional response in macrophages, as evidenced by the upregulation of IL-1β, IL-6, TNF-*α*, and iNOS/NOS2 (cf. Figure 4c), namely, the canonical markers of M1-like activation. In parallel, *Pg*-OMVs increased the expression of REDD1/DDIT4, a stress-response regulator that inhibits mTOR signaling, and GLUT1/SLC2A1, a glucose transporter required for the metabolic shift toward glycolysis during inflammation. Their coordinated induction suggests that *Pg*-OMVs not only trigger inflammatory gene expression, but actively reshape macrophage metabolism to favor a glycolytic, low-OXPHOS state. This is a novel finding because it directly links *Pg*-OMVs to transcriptional control of the mTOR–glycolysis axis, indicating that *Pg* manipulates macrophage bioenergetics at the gene-regulatory level rather than solely through direct mitochondrial inhibition. Such metabolic rewiring may play a critical role in immune evasion by creating macrophages that are inflamed yet metabolically constrained and therefore less capable of executing effective antimicrobial responses.

Regarding the above item (iv) and according to microscopy observations (cf. Figure 1), macrophages appeared similarly activated by both LPS and *Pg*-OMVs. The profound molecular, structural, and metabolic remodeling, which involves lipids, proteins, nucleic acids, and carbohydrates, visually reflected in enhanced membrane roughness and formation of cell agglomerations. Small surface droplets also appeared due to lipid remodeling, hinting to a dramatic reorganization in the cell lipid structure. Recent studies have shown that defects in mitochondrial respiratory chain activity can raise intracellular free cholesterol via altered endoplasmic reticulum/mitochondria communication, changes in sterol sensing and trafficking, and compensation through lipid metabolic programs (Wall et al., 2022; Goicoechea et al., 2023). In other words, an impaired electron transport chain can reprogram sterol metabolism, thus producing net cholesterol accumulation. Moreover, *Pg*-OMVs themselves carry sphingolipids and deliver them to host cells, which provides a direct route to increased host sphingomyelin levels in macrophages after *Pg*-OMV exposure (Sartorio et al., 2022; Jiang and Huang, 2024). Historically, *Pg* and its OMVs have been shown to induce macrophage foam-cell formation and cholesterol-ester droplet accumulation (especially in the presence of lipoprotein cholesterol), which matches the observed rise in cholesterol species after OMV treatment (Qi et al., 2003). Regarding the connection between mitochondrial impairment and an altered sphingolipid metabolism, mitochondrial dysfunction influences sphingolipid enzymes (i.e., sphingomyelinase and ceramide metabolism) and mitochondrial pools of sphingomyelin/ceramide are known to affect cytochrome *c* release and membrane physical properties. Conversely, changes in sphingomyelin/ceramide can alter mitochondrial membrane function and apoptotic signaling. In other words there is a two-way crosstalk, which can produce a feedback loop (Birbes et al., 2005; Novgorodov et al., 2019).

Regarding lipid metabolism, Raman spectroscopy revealed that the presence of *Pg*-OMVs induced a more than twofold increase in macrophage lipid signals compared to LPS (cf. Figure 8b), highlighting a qualitatively distinct metabolic impact. This amplified lipid signature likely reflects both direct deposition of bacterial lipids and host-driven lipid remodeling. *Pg*-OMVs deliver outer-membrane phospholipids, lipoproteins, and gingipains directly into macrophages via fusion or endocytosis, introducing exogenous lipids that LPS alone cannot provide (Kuehn and Kesty, 2005; Veith et al., 2014). In parallel, *Pg*-OMVs trigger broader intracellular signaling than LPS by activating both TLR2 and TLR4 pathways (Darveau et al., 2004; Jain and Darveau, 2010), and inducing metabolic regulators, including REDD1 and GLUT1 (Seyama et al., 2020). Interestingly, the upregulation of REDD1/DDIT4 and GLUT1/SLC2A1 was only modest compared with LPS stimulation, likely reflecting partial saturation of stress- and glycolysis-related pathways by LPS, a hybrid M1/M2-like metabolic phenotype induced by *Pg*-OMVs, or preferential reprogramming toward lipid metabolism rather than additional glycolytic flux, consistent with the pronounced lipid accumulation detected by Raman spectroscopy. Even modest induction of REDD1 and GLUT1 indicates engagement of mTOR-inhibitory and glucose-uptake pathways that complement the inflammatory program. The novelty here lies in the direct, label-free detection of lipid accumulation in macrophages in response to *Pg*-OMVs and the demonstration that OMVs can simultaneously induce a strong inflammatory response while selectively rewiring metabolic pathways. Previous studies characterized OMV-induced cytokine production, while *Pg*-OMVs also uniquely drive lipid remodeling and partially decouple glycolytic and mitochondrial responses, mechanisms hardly captured by conventional assays. The resulting lipid accumulation supports membrane remodeling, lipid droplet formation, and inflammatory mediator synthesis with constrained oxidative metabolism, thereby creating macrophages that are inflamed yet metabolically constrained (Bozza and Viola, 2010; O’Neill and Pearce, 2016).

Finally, the important hint obtained by flow cytometric analyses indicated that both LPS and *Pg*-OMVs promoted the activation of RAW264.7 cells, as reflected by increased granularity and elevated expression of CD86 and MHC-II (Liu et al., 2022). However, the stronger shift observed in *Pg*-OMV-treated cells suggests that vesicle-associated components may engage additional pattern-recognition pathways or be internalized more efficiently, resulting in enhanced intracellular remodeling and antigen-presentation capacity. This supports the hypothesis of *Pg*-OMVs as strong immunostimulatory units, driving macrophage activation to a degree comparable to classical TLR agonists.

Collectively, these findings identify a previously underappreciated mechanism by which *Pg*-OMVs reprogram macrophage bioenergetics and lipid metabolism, providing novel insight into bacterial immune evasion strategies in chronic periodontal inflammation.

### 4.2 Manipulation of macrophage metabolism by Pg-OMVs

Macrophage functional polarization is tightly orchestrated by metabolic reprogramming, with M1 and M2 states representing two ends of a dynamic spectrum shaped by environmental cues. Classically activated M1 macrophages rely predominantly on aerobic glycolysis, exhibit disrupted tricarboxylic acid cycle activity, accumulate metabolites such as succinate that stabilize HIF-1*α*, and display reduced mitochondrial oxidative phosphorylation, a configuration that supports rapid adenosine triphosphate generation and pro-inflammatory effector responses (O’Neill and Pearce, 2016; O’Neill et al., 2016; Stunault et al., 2018). In contrast, M2 macrophages, associated with tissue repair, resolution of inflammation, and immune regulation, depend on intact oxidative phosphorylation, fatty acid oxidation, and a functional tricarboxylic acid cycle, with specific pathways playing a pivotal role in reinforcing their anti-inflammatory program (Huang et al., 2014; Liu et al., 2017). A review of the so far published literature points to key evidences indicating that *Pg*-OMVs can exploit both the above metabolic checkpoints to manipulate macrophage functions (Cecil et al., 2017; Castillo et al., 2022). Several studies demonstrated that *Pg*-OMVs drive macrophages toward a glycolytic, M1-like metabolic signature, including suppressed mitochondrial respiration, enhanced lactate production, and upregulation of glycolysis-related enzymes, accompanied by elevated secretion of IL-1β, IL-6, TNF-α, and nitric oxide (Fleetwood et al., 2017; Cecil et al., 2017). Such a metabolic shift mirrors the pathogen-associated activation commonly induced by toll-like receptor ligands (e.g., LPS, as shown here) that activate the immune system, and represents an early innate immune defense response. Intriguingly, however, *Pg* can also interfere with pathways required for M2 polarization, such as by limiting the availability of vital metabolic intermediate or altering lipid mediators needed for sustaining oxidative phosphorylation, thereby hindering the acquisition of a fully regulatory phenotype (Zhang S. et al., 2025; Zhang Z. et al., 2025; Luo et al., 2024). Such a strategy may create a biphasic or “stalled” polarization state, in which macrophages exhibit initial pro-inflammatory metabolic activation but fail to transition into a resolving, M2-like state, ultimately supporting chronic inflammation while simultaneously enabling immune evasion and persistence of the pathogen within host tissues. Thus, *Pg*-OMVs appear capable of inducing sequential and metabolically distinct macrophage states: an acute glycolytic burst characteristic of M1 activation, followed by partial metabolic rewiring that impairs full M2 differentiation. This dual-phase manipulation of macrophage immunometabolism aligns with emerging models of periodontal disease pathogenesis and highlights immunometabolic pathways as potential therapeutic targets for controlling *Pg*-associated dysbiosis and chronic inflammation (Luo et al., 2024).

**Figure 13:**
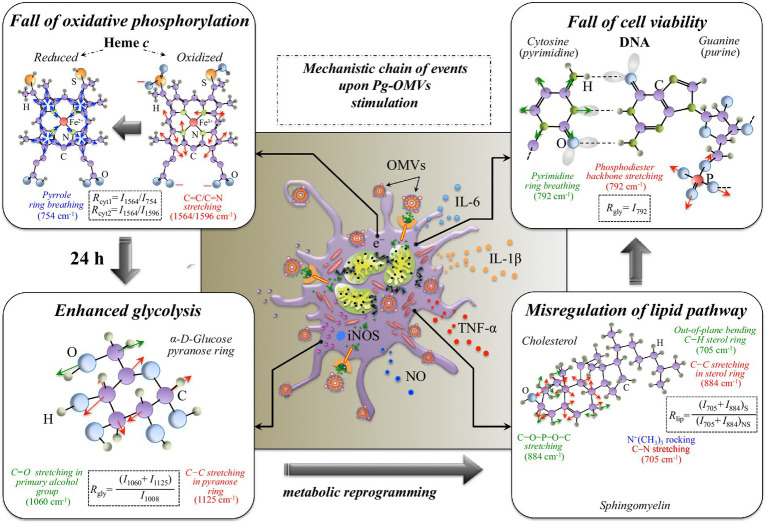
Schematic draft summarizing the effects of *Pg*-OMVs stimulation on RAW264.7 cells and the Raman markers and related parameters proposed in this study to make a quantification of cells’ metabolic disturbances by *Pg*-OMVs.

The present data point to a reduced oxidative phosphorylation together with increased glycolysis, which are both hallmarks of the M1 pro-inflammatory phenotype. An important detail, revealed by flow cytometric analyses, was that macrophages stimulated by *Pg*-OMVs underwent stronger shifts than in the case of LPS stimulation (cf. Figures 4d,e). This is clearly connected with the fact that *Pg*-OMVs contain not only LPS but also other toxins that strongly shift macrophages toward activation and glycolytic metabolism. However, the Raman spectroscopic analysis of cytochrome *c* redox bands revealed that macrophages exposed to LPS or *Pg*-OMVs maintained a significantly more reduced cytochrome *c* pool compared to unstimulated controls. Although a gradual increase in oxidation occurred over 24 h in all conditions, the persistent attenuation of cytochrome *c* oxidation in the stimulated groups indicates a sustained impairment or inefficiency of mitochondrial electron transport activity. Such a redox pattern is consistent with a partial inhibition of complexes I and IV, namely, the entry and exit points of the respiratory chain (Musatov and Robinson, 2012). This is a well-established consequence of inflammatory signaling and inducible nitric oxide synthase activation in macrophages when stimulated by pathogen-associated molecular patterns (e.g., LPS, lipoproteins, and peptidoglycan fragments) (Goulopoulou et al., 2016). This metabolic signature, characterized by reduced mitochondrial oxidation and a compensatory reliance on glycolysis, is emblematic of an M1-like pro-inflammatory state. Notably, the temporal softening of this inhibition in *Pg*-OMV–treated macrophages, while still remaining below control levels, may reflect a non-canonical or hybrid polarization state in which an initial M1-like metabolic shift is tempered by bacterial immune-evasion mechanisms that promote partial metabolic recovery. Thus, *Pg*-OMVs appear to drive a metabolic trajectory that begins with classical inflammatory activation and yet diverges toward a dampened or regulatory phenotype over time, rather than a full M2-type oxidative program. Figure 13 schematically summarizes the overall metabolic changes as induced by *Pg*-OMV stimulation on macrophages with related Raman markers and quantitative spectroscopic parameters, as determined in the present study.

## 5 Conclusion

Raman spectral analysis indicated that *Pg*-OMVs elicited an early metabolic shift in macrophages characterized by partial inhibition of mitochondrial oxidative activity and a successive compensatory increase in glycolytic flux. An increased intensity in glucose-ring Raman bands, as observed in *Pg*-OMV-stimulated macrophages in an extent similar to the case of LPS stimulation, confirmed the expression of an enhanced glycolytic phenotype. This, in turn, reflects an elevated glucose uptake and successive accumulation of glucose-derived metabolites that accompany pro-inflammatory metabolic reprogramming. These patterns are consistent with the M1-like pro-inflammatory metabolic reprogramming typically associated with innate immune activation. Such transient glycolytic activation has already been reported as part of the host response to *Pg* and other immune-evasive pathogens. However, an attenuated oxidation of cytochrome *c*, as revealed by Raman spectroscopy, and the persistent inhibition of mitochondrial electron transport observed in *Pg*-OMV-stimulated macrophages can be interpreted as part of the immune-evasion strategy employed by *Pg*. Upon suppressing complex I and IV mitochondrial activities and enforcing a glycolytic bias, *Pg*-OMVs drive macrophages into a metabolically dysregulated inflammatory state that is insufficiently oxidative to support efficient bactericidal activity. The partial metabolic recovery at later time and without restoration of full mitochondrial functionality further suggests a shift toward a hybrid or regulatory phenotype that favors bacterial persistence. Together, these metabolic signatures support the interpretation that *Pg* modulates macrophage bioenergetics as a survival strategy. The observed metabolic activation path may precede or contribute to a later regulatory (M2-like) phenotype that pathogens exploit in the long-range to attenuate host defense. Accordingly, *Pg*-OMVs appear to drive a biphasic macrophage response, an initial glycolytic, pro-inflammatory signature, possibly followed by metabolic features supportive of immune modulation and bacterial persistence.

This study shows that Raman spectroscopy can provide time-lapse, label-free insights into macrophage metabolic reprogramming, revealing mitochondrial and glycolytic dynamics that complement conventional immunological assays. The strong concordance between Raman spectroscopy and immunological assays makes Raman analysis as a primary candidate to reliably delineate macrophage responses to LPS, *Pg*-OMVs and other stressors. Provided that future studies will confirm the present Raman approach to immunology issues, Raman spectroscopy could soon reach, in many practical cases, the stage of functioning as a standalone analytical platform versus a series of complementary techniques, with considerable time and economic savings.

In conclusion, this study confirms that *Pg* is not only the key bacterium in the progression of periodontitis, but also a potential pathogen capable to promote systemic inflammation through its OMVs. In particular, the combined impact of toxins included in *Pg*-derived OMVs could contribute to neuroinflammation in Alzheimer’s-like pathologies and be possibly involved also in chronic inflammation-driven peripheral diseases such as atherosclerosis and rheumatoid arthritis.

## Data Availability

The original contributions presented in the study are included in the article/Supplementary material, further inquiries can be directed to the corresponding author.
