# Supplementary material for: Deciphering macrophage molecular responses to Porphyromonas gingivalis outer membrane vesicles through combined immunoassay and Raman spectroscopic analyses
Source: Front Microbiol. 2026 May 29;17:1803640. doi: 10.3389/fmicb.2026.1803640 (PMC13260299; doi:10.3389/fmicb.2026.1803640)
Supplement: Supplementary file 1 [file Table_1.docx]

**Supplementary Information**

**Table S-I:** List of the used reagents, their suppliers, and stock solution preparations.

**Table S-II:** List of the primers used in assessing the relative gene expression as calculated by the ΔΔCt method using Rplp0 gene as an internal control.

**Table S-III:** List of wavenumbers at maximum and vibrational assignments for all deconvoluted bands included in Zone I (references given hereafter).

**Table S-IV:** List of wavenumbers at maximum and vibrational assignments for all deconvoluted bands included in Zone IV (references given hereafter).

**References:**

(1) K. Czamara, K. Majzner, M. Z. Pacia, K. Kochan, A. Kaczor, and M. Baranska, Raman spectroscopy of lipids: a review, *J. Raman Spectrosc.* **46,** 4−20 (2015).

(2) G. Zhu, X. Zhu, Q. Fan, X. Wan, Raman spectra of amino acids and their aqueous solutions, *Spectrochim. Acta Part A: Mol. Biomol. Spectrosc.* **78,** 1187−1195 (2011).

(3) M. Tsuboi, Y. Ezaki, M. Aida, M. Suzuki, A. Yimit, K. Ushizawa, T. Ueda, Raman scattering tensors of tyrosine, *Biospectroscopy* **4,** 61−71 (1998).

(4) E. Wierchigroch, E. Szafraniec, K. Czamara, M. Z. Pacia, K. Majzner, K. Kochan, A. Kaczor, M. Baranska, K. Malek, Raman and infrared spectroscopy of carbohydrates: a review, *Spectrochim. Acta Part A: Mol. Biomol. Spectrosc.* **185,** 317−335 (2017).

(5) F. Madzharova, Z. Heiner, M. Guehlke, and J. Kneipp, Surface-enhanced hyper-Raman spectra of adenine, guanine, cytosine, thymine, and uracil, *J. Phys. Chem.* **120,** 15415−1523 (2016).

(6) S. Hu, I. K. Morris, J. P. Singh, K. M. Smith, and T. G. Spiro, Complete assignment of cytochrome *c* resonance Raman spectra via enzymatic reconstitution with isotopically labeled hemes, *J. Am. Chem. Soc.* **115,** 12446−12458 (1993).

(7) I. Notingher, C. Green, C. Dyer, E. Perkins, N. Hopkins, C. Lindsay, L. L. Hench, Discrimination between ricin and sulphur mustard toxicity *in vitro* using Raman spectroscopy, *J. R. Soc. Interface* **1,** 79–90 (2004).

(8) E. B. Hanlon, R. Manoharan, T.-W. Koo, K. E. Shafer, J. T. Motz, M. Fitzmaurice, J. R. Kramer, I. Itzkan, R. R. Dasari, and M. S. Feld, Prospects for *in vivo* Raman spectroscopy, *Phys. Med. Biol.* *45,* 1–59 (2000).

(9) F. D’Amico, F. Cammisuli, R. Addobbati, C. Rizzardi, A. Gessini, C. Masciovecchio, B. Rossi, and L. Pascolo, Oxidative damage in DNA base revealed by UV resonant Raman spectroscopy, *Analyst* **140,** 1477–1485 (2015).

(10) M. Shanmugasundaram and M. Puranik, Computational prediction of vibrational spectra of normal and modified DNA nucleobases, *J. Raman Spectrosc.* **40,** 1726–1748 (2009).

(11) R. Tuma, Raman spectroscopy of proteins: From peptides to large assemblies, *J. Raman Spectrosc.* **36,** 307–319 (2005).

(12) J. R. Beattie, S. E. J. Bell, and B. W. Moss, A critical evaluation of Raman spectroscopy for the analysis of lipids: Fatty acid methyl esters, *Lipids* **39,** 407–419 (2004).

(13) E. Bicknell-Brown, K. G. Brown, and W. B. Person, Configuration-dependent Raman bands of phospholipid surfaces. 1. Carbonyl stretching at the bilayer interface, *J. Am. Chem. Soc.* **102,** 5486–5491 (1980).

(14) N. Wang, J. Wang, P. Wang, N. Ji, and S. Yue, Label-free Raman spectromicroscopy unravels the relationship between MGMT methylation and intracellular lipid accumulation in glioblastoma, *Anal. Chem.* **95,** 11567–11571 (2023).

(15) I. Hazegawa, Y. Nishina, K. Sato, M. Shikiri, R. Miura, K. Shigat, A Raman study of the C(4)=O stretching mode of flavins in flavoenzymes: Hydrogen bonding at the C(4)=O moiety, *J. Biochem.* **121,** 1147–1154 (1997).

(16) M. Roman, T. P. Wrobel, C. Paluszkiewicz, W. M. Kwiatek, Comparison between high definition FT-IR, Raman and AFM-IR for subcellular chemical imaging of cholesteryl esters in prostate cancer cells, *J. Biophotonics* **13,** e201960094 (2020).

(17) A. Zajac, J. Michalski, M. Ptak, L. Dyminska, A. Z. Kucharska, W. Zierkiewicz, and J. Hanuza, Physicochemical characterization of the loganic acid – IR, Raman, UV-Vis and luminescence spectra analyzed in terms of quantum chemical DFT approach, *Molecules* **26,** 7027 (2021).
